# Practical Notes and Formulæ

**Published:** 1877-09-20

**Authors:** 


### Practical Notes and Formulæ.

PURPURA HÆMORRHAGICA.—By Prof. Troussea, of Paris:

R. Ferri et potassii tartratis........dr.ij
Acidi tartaraci................gr.iij
Syrupi tolutani................oz.i
Aquæ canelæ..................dr.v
Aquæ distillatæ...............oz.iijss M

A tablespoonful every two hours.

CHILL REMEDY. (*Naphey*).—

R. Quinæ Suephatis..........grs.xxx
Olei piperiti nigri. . .....gtt.xxiv
Acidi sulphurici aromat........dr.
Syrupi simplicis..............oz.iv
Alcoholi.........................oz.j M

Tablespoonful every two hours during the intermission, until half the quantity be taken. After which, every three hours, until all be taken.

TO ABORT A CHILL.—If the chill-time is near at hand, and you wish to head it off, may do so by putting patient to bed and administering the following:

R. Chloral hydratis..... .. ....gr.xx
Aquæ camphoræ..............oz.i M

For one dose, he will go to sleep and the sweating stage will ensue at once, as a general thing. If the chill has actually commenced, a dose of opium followed by the inhalation of chloroform, has been known to abort it.

PRESCRIPTION FOR DYSMENORRHEA.—

R. Fl. ext. cyprepedium (ladies' slipper).
Aquæ camph..............aa oz.ij
Bromide potass.............dr.iij M

Dose, two teaspoonfuls three times a day, gradually increasing the dose to a tablespoonful. W.

ARSENIC IN CHRONIC CHILLS, ETC.—The following formula for the administration of arsenic strikes us with much favor as of safe dose for long continuance, and well adapted to chronic chills; also useful in skin affections, in lencorrhea, and as an anti-dyspeptic and tonic pill:

R. Ac. arsenic....................gr.j
Ferri. sulph. exic.............gr.xx
Pulv. capsicum.................dr.j
Pil. al. et myrrh..............q. s.

Ft. pills No. LX.

One pill two or three times a day.

*Another fine tonic is—*

R. Cit. iron et quinine............gr.v

Liq. strichniæ............... m.v

Inf. columbæ...................oz.j

For a dose. Particularly useful in ammorrhea, and in debilitated conditions generally.

---

STRYCHNIA.—Strychnia is coming into such general use as a tonic, anti-dyspeptic, etc., that it is well to give formulæ for its use. It does not dissolve readily in alcohol, and hence the tinctures used are often of imperfect strength. The following formula gives a thorough solution, and will be found available for all purposes where strychnine is indicated:

R. Strychniæ acetatis.............gr.i
Acidi acetici.................m.xx
Alcoholis ....................dr.ij
Aquæ..........................dr.vj M

Dose, 10 drops containing about 1-40th grain.

---

GONORRHEA.—Dr. M. D. Mooney, of Georgia, (in *Jour. Mat. Med.*,) reports fine success in the treatment of gonorrhea with the following

R. Sugar of milk...................dr.ss
Ext. Indian cannabis....... gr.xx

Mix thoroughly, and divide into sixty powders, one to be taken every three or four hours. The cure is effected in ve to seven days.

---

FOR BRONCHITIS AND ASTHMA.—

R Tinc. lobelia.................

Camphor water.............aa oz.ss

Muriate of morphine..........gr.ij

Tinc. bloodroot................oz.i

Empyreumatic syrup..........oz,vj M

Half to one dram every three hours. Well adapted to coughs, when the secretions are tough and the expectoration scant and painful. W.

---

RHEUMATISM (*Recipe for*).—Joseph Lomax, M. D., Troy, N. Y., has found the following formula very valuable in the treatment of acute rheumatism:

R. Salicylic acid................dr.iij

Bicarb soda..................dr.ij

Glycerine ................ } aa ozij

Water..................... } M

Tablespoonful every two hours the first day, afterward a dose every six hours.

---

IRRITABLE BLADDER FROM ACID URINE. (*Naphey*).—

R. Uvæ ursi folia...............oz.iss
Humuli folia.................oz.ss

Pour on a quart of boiling water, and after two hours, add—

Sodæ bicarb .................dr.ij
Morphine.....................gr.ij

Dose, a tablespoonful frequently.

---

GOOD, CHEAP PASTE.—

R. Dextrine.....................2 oz.

Acetic acid ............ } aa 4 oz.

Alcohol................. }

Water..... ................2½ oz.

Mix the dextrine, acetic acid and water, stirring until thoroughly mixed; then add the alcohol.—*Brief.*

---

COLD IN THE HEAD.—

R. Murate of morphia............2 gr.
Light carbonate of bismuth... 6 dr.
Starch......................2 dr. M

Of the powders, one-quarter to one-half may be taken as snuff in the twenty-four hours, for influenza, cold in the head, neuralgia, etc.—*Brief.*

---

DELIRIUM TREMENS.—A good anodyne in delirium tremens is the following:

R. Chloroformi.................dr.ss
Sulph. quinine................gr.ij
Tinc. cardamomi. com........dr.j
Aquæ camph...................dr.x

For one dose.

---

NUTRITIVE ENEMAS.—In cases where nourishment cannot be administered by the mouth, life has been prolonged, and the patient sometimes saved, by nutritive enema. The following recipe (from *Dunglison's* late work) will be found convenient for this purpose:

*Beef Tea and Cream Enema.*—Mix together—

R. Strong beef tea.................4 oz.

Cream .. .....................1 oz.

Brandy................. ... ½ oz.

Port wine.....................½ oz.

May be used three times a day. If brandy is not indicated, take beef tea, soup, or milk, and eggs beaten together and thicken with corn flour.

*Cod Liver Oil and Bark Enema.*—

R. Milk.........................4 oz.

Port wine... .... .. ........1 oz.

Cod liver oil...................½ oz.

Tinc. of yellow bark..........2 dr.

Liquid ext. of opium........20 gtt.

---

In Hemoptysis.—

R. Fluid ext. ergot....

Tinc. opii camphorat } equal parts.

Syrupi tolutan......

A desertspoonful every half hour.—*Brief.*

---

Conium Maculatum.—This remedy is not sufficiently appreciated by the profession. It is a narcotic of great power, though often inert as found in the shops —particularly so with the solid extract. It does not, like opium, interfere with the nutritive or secretory functions, and is not cumulative in its effects, and may, therefore, be administered for a long period with safety. In chorea, in syphilitic rheumatism, in rheumatic neuralgia, in asthma attended with painful respiration, it is a valuable agent. Combined with opium, it modifies its effects and, to some extent, corrects its deleterious properties. For this latter purpose, the following formulæ will be found useful, and will be borne by patients who can not take opium alone:

R. Fluid ext. conium............

Deod. tinc. opii........... ..aa oz.j M

Dose, 10 to 30 drops.

R. Fluid ext. conium............oz.ss
Aquæ camph.... ............dr.ij
Muriati morph................gr.j M

Dose, one teaspoonful.

R. Fluid ext. conium ...... ...oz.ss

Sp'ts nitre dulc.............

Camph. tinc. opii...........aa oz.j M

Dose, one teaspoonful. A good substitute for Dover's powder, and specially useful as an anodyne in typhoid pneumonia. W.

---

Excellent Hydrogogue Cathartic.

R. Podophylin ..................grs.v
Bitartrate potassa.. ..........oz.ss

Mix thoroughly and divide into ten powders, one to be given every three hours. Valuable in dropsical accumulations. Given every third or fourth day, with ten drops of tinc, digitalis, thrice daily in the interval, will be found an excellent treatment in hydrothorax and other forms of dropsy. W.

---

Trismus Nasceitium.—Dr. J. N. Nixon, Springfield, Ind., reports to the *Ohio Medical Record* two cases of trismus nasceitium successfully treated by repeated revulsive applications of chloroform to the spine. A soft, cotton cloth was saturated in chloroform and applied to the entire length of the spine, covering it with oil silk to prevent evaporation. The application irritates severely, and will vesicate if long continued. On removal, the pain quickly subsides, and the patient falls asleep.

---

Arnica—Poisonous.—The tincture of arnica, as a local application to sprains and bruises, has long been extensively used. It has become a favorite, especially among the homeopaths, and by consequence amongst a certain class of stylish or fashionable people. We have never thought that it possessed any remarkable advantage as a liniment other than what is contained in the alcohol, with which the tincture is prepared. Of late, a number of cases have been reported of poisonous effects upon the skin from its use.

---

Incontinence of Urine in Children.—*Bromide of Iron* is said to be a very reliable remedy for incontinence of urine, especially in children.

VINEGAR IN OPIUM POISONING.—Dr. J. D. Fawcett (in *Med. Brief*) recommends the use of vinegar as an antidote to opium, to be given freely internally, and the body sponged with it externally. He reports two cases thus relieved. In the one forty grains of opium had been taken, and the patient in a stupor when the doctor arrived. Six ounces of vinegar were given, and the body rubbed with it, and relief followed in half an hour. In the other, patient had swallowed ten grains of morphine, and was promptly relieved by two wine-glasses full of vinegar.

---

OPIUM POISONING.—Give at once a full emetic dose of sulphate of zinc, followed by warm coffee, containing ipecac, to be freely and repeatedly given. Inject also, hypodermically, the thirtieth or fortieth of a grain of the sulphate of atropia, and repeat at intervals of twenty minutes if necessary. If free emesis is produced, little fear need be apprehended. The dilatation of the pupil will indicate how far to push the atropia.

